# Train Traffic as a Powerful Noise Source for Monitoring Active Faults With Seismic Interferometry

**DOI:** 10.1029/2019GL083438

**Published:** 2019-08-26

**Authors:** F. Brenguier, P. Boué, Y. Ben‐Zion, F. Vernon, C.W. Johnson, A. Mordret, O. Coutant, P.‐E. Share, E. Beaucé, D. Hollis, T. Lecocq

**Affiliations:** ^1^ ISterre, Université Grenoble Alpes Gières France; ^2^ Department of Earth Sciences University of Southern California Los Angeles CA USA; ^3^ IGPP University of California, San Diego La Jolla CA USA; ^4^ EAPS MIT Cambridge MA USA; ^5^ Sisprobe Grenoble France; ^6^ Royal Observatory of Belgium Brussels Belgium

**Keywords:** earthquakes monitoring, seismic interferometry, vehicle traffic seismic noise, body waves

## Abstract

Laboratory experiments report that detectable seismic velocity changes should occur in the vicinity of fault zones prior to earthquakes. However, operating permanent active seismic sources to monitor natural faults at seismogenic depth is found to be nearly impossible to achieve. We show that seismic noise generated by vehicle traffic, and especially heavy freight trains, can be turned into a powerful repetitive seismic source to continuously probe the Earth's crust at a few kilometers depth. Results of an exploratory seismic experiment in Southern California demonstrate that correlations of train‐generated seismic signals allow daily reconstruction of direct *P* body waves probing the San Jacinto Fault down to 4‐km depth. This new approach may facilitate monitoring most of the San Andreas Fault system using the railway and highway network of California.

## Key Points

- Freight trains in Southern California are locally equivalent to a magnitude 2.2 earthquake every day
- We use train noise to reconstruct repetitive virtual sources of *P* waves crossing the San Jacinto Fault at 4‐km depth
- Predictions of train noise across California show potential for passive monitoring of most of the San Andreas Fault system

## 1 Introduction

Recent observations of early deformation phases preceding moderate to large earthquakes (Bouchon et al., 2011; Kato et al., 2012; Niu et al., 2008) raise the hope that real‐time, continuous monitoring of fault zones will allow improved earthquake forecasting. Scuderi et al. (2016) observed precursory changes of seismic wave speed in laboratory faults for the complete spectrum of failure modes found for tectonic faults. Despite considerable efforts to monitor seismic wave speed on real faults (Niu et al., 2008; Tsuji et al., 2018), the prohibitive cost of operating permanent sources of seismic waves that can continuously probe seismogenic sections of active fault zones impedes the ability to directly monitor seismic velocity changes at depth.

Seismic interferometry concepts (Campillo & Paul, 2003) related to the emergence of the Green's function in correlations of a diffuse field led to useful applications in seismology. The cross correlation of seismic noise generated by extended sources in space and recorded at two sensors were shown to provide the impulsive response of the Earth between the two sensors as if a virtual source was emitted at each of the sensors. This approach proved successful for the reconstruction of low‐frequency surface waves (0.1 to 1 Hz) originating from ocean swell activity (Shapiro & Campillo, 2004), but the reconstruction of high‐frequency (HF) body‐waves (>1 Hz) has been difficult because surface waves mostly dominate seismic noise records.

Reconstructing HF body waves is of major interest because they have a sharp sensitivity to seismic velocity perturbations at seismogenic depths. Studies of Roux et al. (2005), Nakata et al. (2015), and Nakata et al. (2016) showed that, under specific virtual source and receiver configurations involving dense seismic arrays, it is possible to reconstruct body waves traveling in the shallow crust. These arrays are specifically designed for seismic interferometry so that the noise correlations between different pairs of sensors can be stacked along distance bins to increase the signal to noise ratio of the reconstructed Green's function. For HF (>1 Hz) body wave reconstruction, these arrays have a typical interstation distance ranging from 50 to 300 m and can extend up to a few tens of kilometers (Brenguier et al., 2015; Nakata et al., 2015). The noise source for reconstructing these body waves, likely related to ocean shore break, wind, or cultural activity remains unclear. Theoretical (Boschi & Weemstra, 2015) and numerical (Colombi et al., 2014) studies examined how anisotropic noise source distribution that is typically the case on Earth can alter the reconstruction of Green's functions from noise correlations. They illustrate how reconstructing specific ballistic waves, such as direct body waves, does not necessarily require uniformly distributed noise sources but rather noise sources located in specific, limited regions referred to as stationary phase zones. In the following, we describe an application of this theory to the reconstruction of body waves at crustal depths. We show that vehicle traffic, which is one of the most energetic, permanent, anthropogenic seismic source, can be used to reconstruct HF body waves traveling across the San Jacinto Fault in Southern California down to a few kilometers depth. We further illustrate the potential for performing similar studies over much of California using simple model calculations.

## 2 Equivalent Magnitude and Spectral Characteristics of Train‐Radiated Seismic Energy

Freight trains were recognized before as potential noise sources for seismic interferometry, but their study was limited to shallow surface applications (Nakata et al., 2011; Quiros et al., 2016). Inbal et al. (2018) report clear observations of seismic signal generated by freight trains in the Coachella Valley at long distances of tens of kilometers from the railways, providing evidence for the high level of seismic energy radiated by such moving vehicles. In this section we quantify the power of such sources by inferring an equivalent earthquake magnitude for moving trains.

The elastic energy radiated to the ground by vehicles and trains is dominated by a dynamic loading/unloading effect that can be represented as a moving vertical force at each contact point between the vehicle and the ground (L. Li et al., 2018). For earthquakes, the potency *P*_0_ represents the product of slip on a fault (*u*) and the rupture area (*A*) (Ben‐Zion*,* 2003).

(1) $$P_{O}=u\times A.$$

In the case of moving vehicles, we can define the equivalent potency as the product of the elastic vertical displacement below each contact point and the total contact area. The total contact area can be estimated by *W* × *l* with *W*, the width of each contact (typically a wheel) and *l*, the length of a portion of road or railway on which the vehicle or train moves. The vertical displacement (*u*_vert_) induced by the loading of each wheel can be estimated using explicit expressions for solving the Love's problem (D'urso & Marmo, 2013). The total potency of one moving vehicle is then given by multiplying the potency associated with a single wheel with the number of wheels (*N*):

(2) $$P_{O\;\text{vehicle}}=u_{\text{vert}}\times W\times l\times N.$$

The equivalent magnitude of a moving vehicle can be estimated using the empirical scaling relation between potency and local magnitude *M*_*l*_ (Ben‐Zion & Zhu, 2002):

(3) $$M_{l}=\log\left(P_{O}\right)+0.72,$$

with potency in units of cubic meters. In the following section, we apply this approach to study train‐radiated energy for seismic interferometry at a crustal scale and show that, locally, a daily traffic of freight trains can reach an equivalent magnitude >2.

The equivalent magnitude approach allows to quantify the power of train‐radiated seismic energy traffic, but most important for crustal‐scale monitoring is the spectral content of such radiated body waves. Indeed, at too high frequencies (>10 Hz), ballistic body waves are mostly scattered at a crustal scale and cannot be used for measuring proper travel time perturbations. At too low frequencies (<1 Hz), wavelengths of tens of kilometers are too large to detect localized, fault‐related, velocity perturbations. For crustal‐scale monitoring at a few kilometers depth, we are essentially interested in the frequency band [1–10] Hz (Tsuji et al., 2018).

In order to study these spectral characteristics, we performed a numerical study of the seismic wavefield radiated by a typical freight train found in California being 1 km long (Ltrain), moving at 25 m/s (90 km/hr, Vtrain) and composed of cars of 16 m long (L). We again consider that the radiated seismic energy is dominated by a dynamic loading/unloading effect that can be represented as a moving vertical force applied by each wheel on the ballast (L. Li et al., 2018). We model the train load as discretized vertical forces. Each force experiences a source time function modeled by a Dirac comb of period *T* = *L*/Vtrain = 0.64 s (1.5 Hz) and length Ltrain/Vtrain = 40 s. This source time function is delayed for each discretized force by a time needed to account for the moving train. The Fourier transform of the vertical velocity recorded by a receiver located in the middle of the segment at a depth of 500 m clearly exhibits frequency peaks related to the harmonic content of the periodic source with a fundamental frequency of 1/*T* = 1.56 Hz, and overtones at 2/*T* = 3.12 Hz, 3/*T* = 4.68 Hz and higher. The train traffic is an overall broadband seismic source due to the impulsive nature of the load of a wheel on a given portion of a railway. However, it radiates prominent seismic energy above 1 Hz due to the harmonic nature of the repetition of this load at regular times governed by the velocity of the train and the distance between wheels. This is a simple model that does not account for attenuation but it clearly shows that, under typical moving train conditions, this source radiates a high level of body waves in the frequency band of interest above 1 Hz. These modeling results are in agreement with field observations from Fuchs and Bokelmann (2018) and C. Li et al. (2018). A full description and illustration of modeling results including a comparison with seismic radiations from car/truck traffic will be described in a companion paper.

## 3 Reconstruction of Daily, Crustal *P* Waves Across the San Jacinto Fault

**Figure 1:**
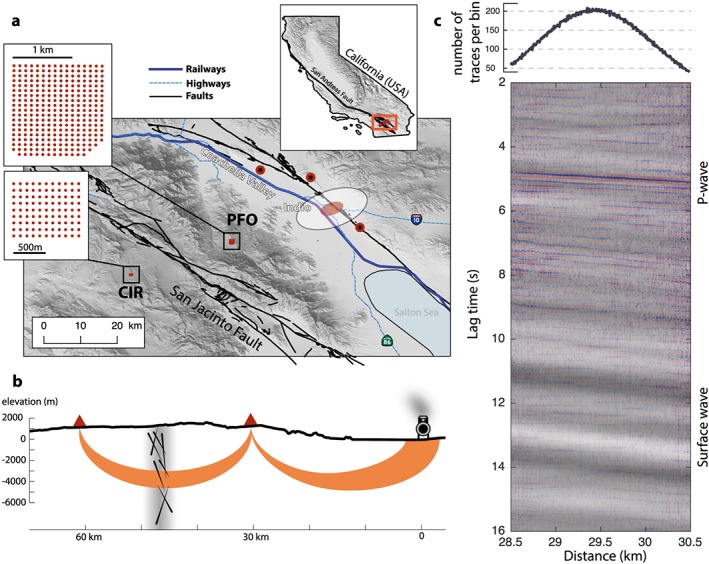
Map of the experiment and virtual seismic section. (a) Location of the two dense seismic arrays in red. The blue line shows the location of the freight train railways and highways in the Coachella Valley. The red and white ellipses show the location of the arrays imprint and stationary phase kernel for the reconstruction of a direct *P* wave between the arrays. The red‐and‐black dots show the location of the seismic stations used on Figure 3. (b) Sketch illustrating the body wave generated by trains and used by seismic interferometry to reconstruct a body wave traveling between the two arrays (in red) across the San Jacinto Fault. (c) Causal and anti‐causal stacked noise correlations between the two arrays using distance‐averaged bins. Each bin is 10 m long. The top figure shows the number of stacked correlations per bin. The high‐frequency content (1–10 Hz) is in color and the low‐frequency content (0.2–1 Hz) in gray. PFO = Piñon Flat Observatory; CIR = Cahuilla Indian Reservation.

We focus in this study on the San Jacinto Fault in Southern California. Based on historic earthquakes and strong strain accumulation, the San Jacinto Fault is believed to pose significant seismic risk in California (Fialko, 2006; Rockwell et al., 2015). A large (*M* > 7) earthquake on the San Jacinto fault could cause major damage in several highly populated southern CA counties (~5 million inhabitants) located at distances of 15 to 150 km from the fault. The trifurcation zone of the San Jacinto Fault (Figure 1) is one of the most seismically active areas in Southern California producing more than 10 % of all earthquakes in this region (Ross et al., 2017).

Our goal is to mimic the approach of Tsuji et al., 2018 for monitoring crustal seismic velocity perturbations but using only correlations of ambient seismic noise. Following a previous study of Brenguier et al. (2015) and Nakata et al. (2016) we designed an exploratory experiment with dense seismic arrays in the San Jacinto Fault region (Figure 1a) with the purpose of using correlations of cultural seismic noise between arrays to retrieve virtual sources of HF (>1 Hz) direct body waves traveling across the fault zone at a few kilometers depth.

Following seismic interferometry principles, the direct *P* wave between two receivers emerges from the correlation between a *P* wave recorded at one receiver and a *PP* wave recorded at the second receiver. The Green's function direct *P* wave reconstruction is exact when the sources of these *P* and *PP* waves are the same or in other words when the *PP* wave recorded at the second receiver reflects at the surface location of the first receiver (Figure 1b). In the ray theory of infinite frequencies, the location of the noise source generating this specific wave propagation geometry is a point at the surface. Its position can be predicted using ray tracing in an accurate velocity model. In practice, using finite frequency waves means that this point becomes an area at the surface referred to as the stationary phase region. It is formally defined as the area where all seismic sources comply with the criteria that the arrival time of the *PP* wave at the second receiver minus the arrival time of the *P* wave at the first receiver is smaller than the arrival time of the *P* wave between the two receivers plus/minus a quarter of the dominant period.

The closest source of cultural seismic noise near the San Jacinto Fault is the Coachella Valley (~40 km from the fault; Figure 1a). Following the study of Inbal et al. (2018), we believe that vehicle traffic is the most energetic source of cultural noise in the valley. In order to evaluate the level of seismic emissions from train and car/truck traffic in the Coachella Valley and following the approach from the previous section 2, we compute the equivalent magnitude of the daily train traffic along a 10‐km‐long portion of freight railways in the Coachella Valley at Indio corresponding to the width of the stationary phase zone (~25 trains per day; USBTS, 2017). A typical, large freight train along this railway weighs on average 7,000 kg/m and is 1 km long. Each car is about 16 m long with a total of about 60 cars per train. Each car has typically two axles with four wheels each (eight wheels total per car). One car weighs 112,000 kg corresponding to a pressure of 28 · 10^6^ Pa applied by each wheel of contact area 0.005 m^2^ (*W* = 0.1 m, *l* = 0.05 m). By solving the Love's problem, we find *u*_vert_ equals 1.3 · 10^−5^ m. We consider that only 1/5 of this deformation is actually transferred to the ground due to absorption from the ballasts. The total contact area of one wheel along a 10‐km‐long portion of railway is (*W* × *l*) =0.1 × 10,000 = 1,000 m^2^. Considering that one train has 500 wheels and a total number of 25 trains per day, we find a cumulative daily potency of

$$P_{O\;\text{train}}=1.3\;10^{-5}/5\times 1,000\times 500\times 25=32.5\;m^{3}$$

Finally, by applying the potency‐magnitude scaling law (3), we find that the equivalent magnitude for 1 day of freight traffic along this 10‐km portion of railways in the Coachella Valley is ***M***_***l***_ ***=* 2.2**. This relatively high value shows that these train‐radiated body waves can likely be detected at few tens of kilometers from the railways, showing promise for crustal applications of seismic interferometry. This is in good agreement with the study of Inbal et al. (2018) that report train tremor observations up to 50 km from the railways.

The equivalent magnitude for vehicle traffic is proportional to the amplitude of the vertical force applied at the surface of the Earth and thus to the cumulative daily tonnage. In the Coachella Valley in southern California where this study focuses, there is an average of 60,000 vehicles (cars and trucks) driving along the Interstate‐10 highway near Indio. This traffic corresponds to a daily tonnage of approximately 144 · 10^6^ kg. It is on the same order of the daily 175 · 10^6^ kg of freight train tonnage used above. Trucks and cars daily traffic along a 10‐km‐long segment of the I‐10 highway thus also represent the equivalent of a magnitude 2.2 earthquake. However, there is likely a significant difference between the spectral characteristics of trains and car/trucks traffic. Indeed, all cars of a train being tied together move at the same velocity with in general the same distance between wheels, which is not the case for the cumulative number of cars and trucks along a 1‐km‐long segment on a highway. Even though it likely contributes to the reconstruction of the observed *P* wave between arrays, highway traffic probably radiates less energy than trains in the frequency band [1–10] Hz, being therefore less effective for crustal imaging and monitoring purposes. This will be investigated in more details in a companion paper.

Following the prediction of strong traffic‐generated noise in the Coachella Valley, we designed the location and separation (30 km) between two arrays (total of 400 seismic stations) located at the Piñon Flat Observatory and the Cahuilla Indian Reservation, so that the noise sources required for the reconstruction of direct *P* waves traveling through the fault at 4‐km depth between the arrays coincides with high‐traffic railways and highways in the Coachella Valley (Figure 1a). We used ray tracing in the velocity model of Allam et al. (2014) to define the distance between arrays (30 km) required to reach a turning point of the ray at 4‐km depth corresponding to the upper seismogenic zone. The red ellipse on Figure 1a corresponds to the location of the noise sources required to reconstruct direct *P* waves between each pairs of receivers of the two arrays in the ray theory (arrays imprint) and the white ellipse to the stationary phase region for the same receiver pairs combinations. At frequencies around 5 Hz, the stationary phase zone at the surface is about 10 km wide and 20 km long. This was obtained using 3‐D ray tracing searching for all sources that comply with the criteria described above. The width of that area is mostly constrained by the distance between the arrays and the velocity model. For example, more homogeneous velocity models produce wider stationary phase area in the radial direction.

Figure 1c presents noise correlations between the two arrays gathered and stacked by distance bins of 10‐m length. This panel corresponds to a 20‐day stack of the correlations of 30‐min‐long whitened noise time series. It shows the stack of the positive and negative parts of the correlation functions filtered in the range 1–10 Hz in color, and 0.2–1 Hz in gray. We observe a clear HF (1–10 Hz) *P* body wave arriving at a travel time of ~5 s, corresponding to an average velocity of ~6 km/s and with an apparent velocity of also ~6 km/s. This is in agreement with the direct *P* wave predicted using full‐waveform modeling and the average velocity model for the area of Allam et al. (2014). By looking separately at the positive and negative parts of the correlation panel (not shown here) we found that the HF body waves are only observable on one side of the noise correlations corresponding to waves traveling from Piñon Flat Observatory (northeast) to Cahuilla Indian Reservation (southwest) in agreement with a source in the Coachella Valley.

**Figure 2:**
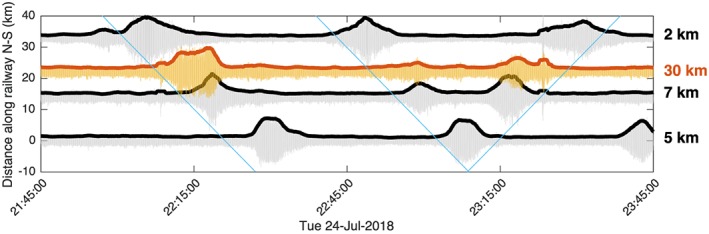
Seismic traces illustrating the passage of trains along the Coachella Valley. Gray signals are filtered signals (1–10 Hz) from seismic stations along the railways (Figure 2), and the yellow signal is the stacked trace for the Piñon Flat array. The blue lines are offset using a velocity of 25 m/s (90 km/hr, 56 M/hr). The numbers on the right show the distance between the stations and the railway. The *y* axis label corresponds to distances of projected seismic station and Piñon Flat array coordinates along the railway.

We further confirm our prediction that trains are among the dominant source of HF body wave noise that contributes to the reconstructed virtual sources. By filtering the raw seismograms of the Piñon Flat array (1–10 Hz) and by stacking all traces with an apparent velocity of 6 km/s for body waves in the direction of the stationary phase zone in the Coachella Valley (white circle in Figure 1), we observe about 25 daily tremor‐like signals lasting about 15 min (yellow trace in Figure 2) that fit very well with the timing of trains running through the Coachella Valley and recorded by seismic stations along the railway (red‐and‐black dots in Figure 1 and gray traces in Figure 2). This proves that the eastern array (Piñon Flat) records a clear body wave signal originating from the train traffic in the Coachella Valley that is likely the main contributor to the reconstructed *P* body waves between the two arrays.

**Figure 3:**
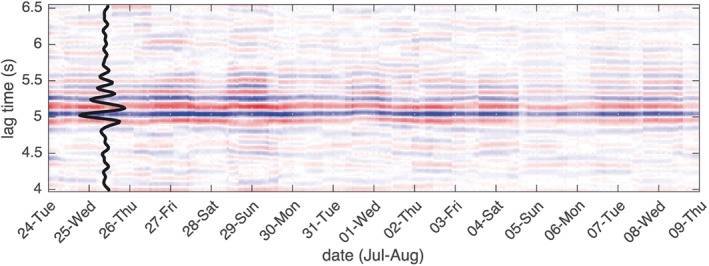
Stacked high‐frequency *P* body wave between the two arrays every 3 hr. The black trace is the stack of all traces.

To study the temporal repeatability of the virtual HF body waves, we compute sections of 3‐hr noise correlations over the 20 days' time span of our data acquisition (July–August 2018). We then slant stack the HF body wave arrival with an apparent velocity of 6 km/s in the direction of the two arrays. Figure 3 shows these 3‐hr‐long stacks that illustrate the high repeatability in time of the virtual body wave source.

## 4 Predicted Level of Train‐Radiated Body Waves Across California

**Figure 4:**
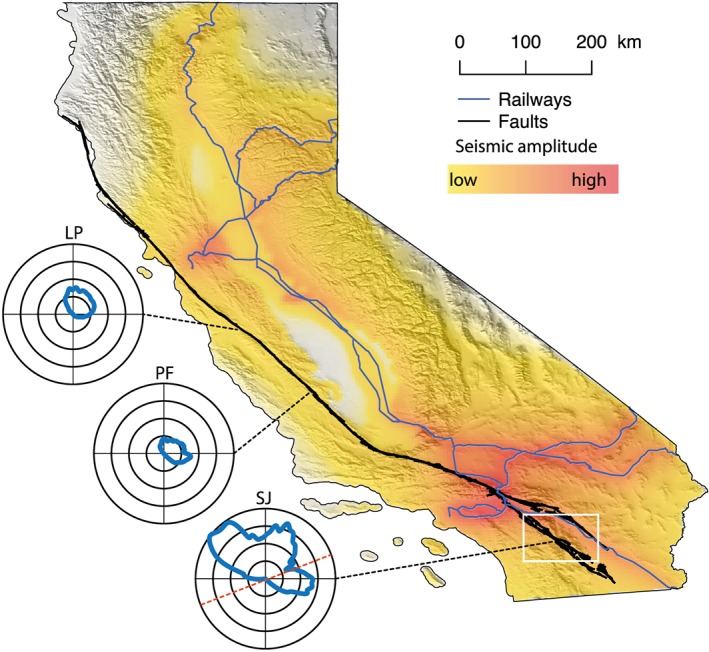
Predicted amplitude of radiated *P* body waves from the major freight train routes of California. The white rectangle illustrates the region of interest for this study shown in Figure 1. The rose diagrams show the amplitude and azimuth of train‐generated body waves at different locations. SJ for San Jacinto Fault, PF for Parkfield, and LP for Loma Prieta. The red dashed line on the SJ rose diagram illustrates the azimuth of the two seismic arrays used in this study.

To explore how freight trains radiated body wave energy across California, we discretize the major freight railways of California as vertical point sources every 2 km (California Department of Transportation, 2013). We consider that each source radiates body waves with an amplitude decay along distance *r* from the source described as

(4) $$A=\frac{A_{0}}{r}\times e^{\frac{-\pi .F.r}{c.Q}},$$

where *A*_0_ is a normalized amplitude at the source defined using relative tonnage of freight trains for different portions of the California railway network [USBTS, 2017], *r* the distance from the source, *F* the central frequency taken as 5 Hz, *c* the *P* wave velocity, and *Q* the quality factor. Our model makes a distinction between hardrock sites and sedimentary basins. We use averaged *P* wave velocity values of 5 and 2 km/s for hardrock locations and sedimentary basin sites and averaged quality factor values of 300 and 50 for hardrock locations and sedimentary basin sites. The calculations use straight rays to estimate *r* and a transmission coefficient of 0.8 for waves traveling from the central valley basin to a hardrock location. Figure 4 shows the cumulative contribution of every individual seismic source attenuated using equation (4) at each model location. The transition from yellow to no color corresponds to the expected level of train tremor detection using a seismometer located in a quiet area according to the observations of Inbal et al. (2018). It roughly corresponds to a distance of 50 km from the railways for a single point source. We believe that this corresponds to the limits of the area within which we can expect to reconstruct crustal body‐waves from daily train‐traffic noise correlations.

Figure 4 demonstrates that train‐radiated body waves can be detected throughout almost the entire state of California and that seismic interferometry using train noise might be used for imaging and monitoring the entire San Andreas Fault system. This level of predicted vehicle traffic noise is probably underestimated because it does not take into account the seismic radiation from trucks and cars on highways. We consider two examples involving locations of the 2004, Magnitude 6 Parkfield and 1989, Magnitude 6.9 Loma Prieta earthquakes. At these locations, rose diagrams of train‐radiated seismic energy show that in the east to west direction the level of radiated body waves is similar to the one predicted for the actual arrays we use in this study. Even though this model does not take into account arrays geometry and the stationary‐phase conditions, it indicates that at these sites we can potentially reconstruct crustal *P* waves in the same way as for the San Jacinto Fault.

## 5 Conclusions and Perspectives

The presented results show that under specific sensor deployment layouts, the seismic waves generated by train and car/truck traffic could be used to reconstruct virtual sources of body waves that can probe the Earth's crust at a few kilometers depth. Additional work is needed to assess how these repetitive virtual sources can be used for monitoring active fault regions with high accuracy. For example, in this study monitoring a 1% velocity change of the direct *P* wave along its entire travel path requires a 0.05‐s accuracy in travel time measurements, which is a quarter of the period at 5 Hz. The stability of the reconstruction of these ballistic body waves versus the length of noise records used for correlations should be studied further to understand how to reach such a level of precision. This novel approach together with new opportunities for recording dense, continuous seismic data (e.g., Distributed Acoustic Sensing) has a strong potential for various applications. These include monitoring temporal changes and imaging the Earth's crust in regions having tectonic earthquakes, induced seismicity, volcanic reservoirs, and geothermal production sites.
